# Virtual patients and standardized patients combined training is associated with improved clinical reasoning among medical students

**DOI:** 10.3389/fmed.2026.1825465

**Published:** 2026-05-11

**Authors:** Tao Shen, Ruitao Zhang, Heng Wang, Dan Li, JiangLi Han

**Affiliations:** 1 NHC Key Laboratory of Cardiovascular Molecular Biology and Regulatory Peptides, Key Laboratory of Molecular Cardiovascular Science, Ministry of Education, Department of Cardiology, Peking University Third Hospital, Haidian, China; 2 Department of Education, Peking University Third Hospital, Haidian, China

**Keywords:** artificial intelligence, clinical reasoning, medical education, standardized patients, virtual standardized patients

## Abstract

**Objective:**

To develop an artificial intelligence (AI)-driven virtual standardized patients (VSPs) system, and to evaluate its educational effectiveness when combined with traditional standardized patients (SPs) training.

**Methods:**

Leveraging natural language processing and a Chinese large language model, we built an AI-powered VSPs application. A total of 80 medical students at Peking University Third Hospital were randomized into two groups: experimental (*n* = 40, VSPs and SPs combined training) and control (*n* = 40, traditional SPs training). The 4-week intervention included assessments on clinical reasoning, core competencies, OSCE performance, and learning experience.

**Results:**

The experimental group showed greater improvement than the control group in clinical reasoning scores (1.3 ± 0.7 vs. 0.3 ± 0.5, 95% CI: 0.73–1.27, *P* < 0.01), core competencies total scores (9.9 ± 4.1 vs. 3.8 ± 2.7, 95% CI: 4.56–7.64, *P* < 0.01), and OSCE performance (11.9 ± 5.2 vs. 3.2 ± 5.0, 95% CI: 6.43–10.97, *P* < 0.01). The improvements were observed in communication, patient care, and internal medicine knowledge. Learner-reported satisfaction was higher (4.6 ± 0.5 vs. 3.2 ± 0.8, 95% CI: 1.10–1.70, *P* < 0.01). Subgroup analysis revealed greater benefits for students with lower baseline scores.

**Conclusion:**

Combined VSPs and SPs training showed better performance than the SPs-only approach used in this study.

## 1 Introduction

Standardized patients (SPs) have been widely adopted in medical education, providing consistency and depth in skills training. However, traditional SPs-based training was costly, resource-intensive, and frequently lacks repeatability and uniformity over time (1–3).

Recent advances in virtual reality (VR) and natural language processing (NLP) have facilitated the development of Virtual standardized patients (VSPs), which offer high consistency, repeatability, and scalability across diverse clinical scenarios (4). Yet, existing VSPs primarily emphasize history-taking while neglecting physical examination interpretation, diagnostic reasoning, and treatment planning—elements that are essential to comprehensive clinical reasoning (5, 6).

Large language models (LLMs), such as ChatGPT and ChatGLM, demonstrate strong semantic understanding and logical reasoning capabilities, creating new opportunities for intelligent simulation in medical education.

To address these gaps, this study introduces an AI-powered VSPs system. The system is built on the XueYiKu application, an artificial intelligence–driven database specifically developed for training and assessing clinical reasoning. The system, when combined with SPs practice, aims to evaluate whether this hybrid approach is associated with better educational outcomes than the SPs-only approach used in this study.

## 2 Materials and methods

### 2.1 System architecture

The clinical reasoning training system consisted of three layers: a data layer, a model layer, and an application layer.

Data layer: integrated multiple medical knowledge sources, including electronic medical records, textbooks, journal articles, clinical guidelines, clinical pathways, expert scoring data, et al. These covered common internal medicine conditions such as hypertension, coronary heart disease, pneumonia, and diabetes. All records were anonymized, cleaned, and structurally standardized in accordance with ethical requirements.

Model layer: developed on large frameworks such as ChatGLM and LLaMA, with fine-tuning and prompt engineering to build a clinical reasoning evaluation model capable of multi-turn interaction, scoring, and feedback.

**FIGURE 1:**
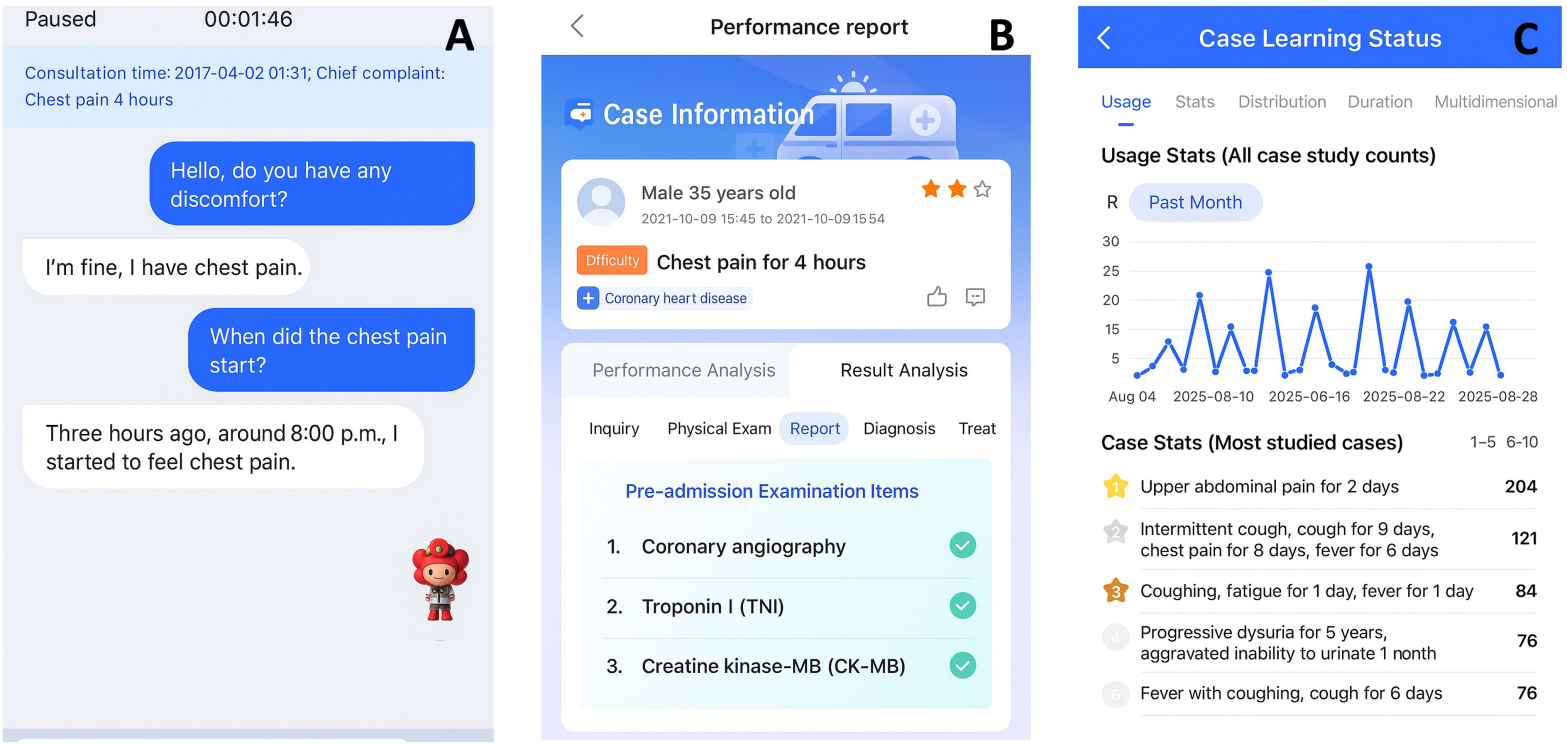
Screenshot of the virtual standardized patients (VSPs) training application interface. **(A)** Interactive dialogue interface for history-taking with the virtual standardized patient. **(B)** Analytical display of the student’s history-taking performance, including structured evaluation and diagnostic feedback. **(C)** Statistical panel summarizing overall learning frequency and case-specific practice counts. The VSPs system provides structured training and feedback to support the development of students’ clinical reasoning skills.

Application layer: implemented through the XueYiKu App, an AI-powered training platform supporting the VSPs module with dialogue simulation, automated scoring, and structured feedback (7). The system adopted a server–frontend separation design with API-level task calls to facilitate model iteration and deployment (Figure 1).

### 2.2 Dataset construction

The raw records underwent data cleaning, terminology standardization, structural analysis, and text completion. Clinical instructors further revised the processed texts. Data augmentation techniques, including back-translation, were applied to create multi-turn dialogue datasets.

The training dataset contained medical records, dialogue texts, clinical reasoning scores, reference completion time, and feedback suggestions. Five assessment dimensions were defined and annotated. Two instructors independently scored each dimension, and the averaged results were used as the final annotation.

### 2.3 Model design and training objectives

Two sub-models were developed: a dialogue generation model and an evaluation model. Supporting modules were also built for the user interface, service calls, and database management. Together, these formed a system capable of multi-turn interaction, multidimensional reasoning assessment, and structured feedback.

The dialogue generation model was trained on multi-turn consultation datasets with supervised fine-tuning. The input included the medical record, previous dialogue turns, and the student’s current inquiry. The output was a simulated patient response. Outputs were reviewed by instructors, and the dataset was iteratively refined through additional fine-tuning and prompt engineering.

The evaluation model was trained with annotated dialogue data. It generated five-dimensional scores, time assessments, and personalized feedback (Table 1). Model performance was assessed using regression metrics such as MAE, RMSE, and R^2^, multi-label classification metrics such as Macro-F1, AUC, and Accuracy@1, and natural language generation metrics including ROUGE-L and BLEU-2. Feedback quality was also validated by expert review.

**TABLE 1:** Design of the diagnostic module based on the virtual standardized patients system.

Dimension	Module content	Task objective	System functionality	Example output	Evaluation method
Information gathering	● History-taking ● Physical examination ● Auxiliary tests	Enable students to comprehensively obtain medical history, physical findings, and essential test results	● Multi-turn dialogue interface with personalized follow-up questions ● Automated standardized physical findings ● Generation of test orders and imaging reports	*“I have had persistent fever for 3 days, worsening cough, and yellow sputum”* *“Moist rales heard in both lungs”* *“Chest X-ray shows patchy opacity in the left lower lung”*	● Information integrity score ● Keyword coverage ● Utilization rate of exam data Accuracy of key test identification
Problem representation	● Case presentation	Enable students to integrate collected information into a concise, logical case description	● Structured input with logical verification ● Prompts on alignment with reasoning conventions	*“Elderly male with fever, cough, and imaging abnormalities, suspected pulmonary infection”*	● Case representation quality score Logical coherence score
Differential diagnosis	● Multiple diagnoses and comparison	Enable students to propose 2–3 differential diagnoses and justify reasoning	● Recognition of diagnostic inputs and rationale ● Evaluation of appropriateness	*“Consider CAP, tuberculosis, bronchitis”*	● Differential diagnosis quality score Coverage and accuracy of prioritization
Justification	● Evidence chain	Enable students to articulate supporting and opposing evidence and construct reasoning chains	● Structured prompts for input ● AI analysis of logical completeness with feedback	*“CAP: supported by fever, imaging opacity, productive cough; TB: not supported due to absence of night sweats and short disease course”*	● Reasoning quality score ● Completeness of reasoning chain ● Proportion of missing reasoning dimensions
Management plan	● Treatment plan ● Comprehensive feedback	Enable students to develop an initial treatment/follow-up plan and receive feedback	● Comparison with guideline-based recommendations ● Automated report generation with visualized feedback	*“Initiate anti-infective therapy* + *hospitalization* + *follow-up imaging”* *“Information collection integrity 1.8/2, case representation 1.4/2*…”	● Appropriateness of management plan score ● Overall five-dimension score ● Personalized improvement suggestions

The model comparison in this study was mainly conducted to support system deployment and feasibility assessment for educational use, rather than as a formal benchmark study of large language models. For this internal comparison, all candidate models were tested on the same task set and prompt framework used for the teaching platform. The comparison focused on practical deployment-related criteria, including scoring accuracy, structural recognition, feedback quality, and response efficiency. This component was intended to inform model selection for educational implementation rather than to serve as a formal head-to-head benchmark of large language models.

### 2.4 Intervention design

Participants were recruited and followed up from April 2022 to December 2022. Eighty internal medicine students from Peking University Third Hospital were randomly assigned in a 1:1 ratio to the experimental group or the control group using a computer-generated random sequence. Group assignments were concealed until enrollment was completed and baseline assessment had been finished. Because this was an educational intervention conducted in a real teaching setting, participants and instructors could not be fully blinded to group allocation; however, standardized scoring criteria and rubrics were used to reduce assessment bias.

No participants were lost to follow-up or excluded. The experimental group (*n* = 40) received combined VSPs and SPs training. Students completed weekly VSPs sessions with automated scoring and feedback, followed by SPs consultations and instructor comments. The control group (*n* = 40) received traditional SPs training, including weekly SPs consultations and collective feedback.

### 2.5 Evaluation metrics

The evaluation framework addressed both model performance and educational effectiveness. Pre- and post-intervention assessments included clinical reasoning tests, core competencies, OSCE examinations, and a brief learner-reported satisfaction questionnaire.

Clinical reasoning scores were assessed using the clinical reasoning assessment Framework (6). This instrument assigns a total of 10 points across five dimensions: information gathering, problem representation, differential diagnosis, justification, and management plan. Clinical reasoning scores were independently rated by two trained clinical instructors using predefined scoring criteria. Inter-rater reliability was assessed using the intraclass correlation coefficient (ICC), which was 0.87 for the total clinical reasoning score, indicating good agreement between raters. Discrepancies were subsequently resolved by discussion for final scoring.

OSCE performance, with a maximum score of 100 points, was measured using a structured, multi-station examination. Each station typically lasted 10 min and assessed history taking, physical examination, reasoning, skills, and communication. The OSCE is internationally recognized as the gold standard for evaluating clinical competence (8). OSCE performance was assessed by trained examiners according to standardized scoring rubrics. Inter-rater reliability was assessed using the ICC, which was 0.84 for the total OSCE score, indicating good agreement. To improve reliability, the same rubric and scoring standards were applied across all stations and both groups.

Core competencies, also scored out of 100 points, were evaluated using a 24-item scale adapted from the ACGME Milestones (9, 10). The scale covered professionalism, communication and collaboration, teaching ability, lifelong learning, knowledge and skills, and patient care. It demonstrated excellent construct validity (KMO = 0.968, Bartlett’s test *P* < 0.01) and reliability (Cronbach’s α = 0.964).

Student satisfaction was assessed using a brief 3-item post-intervention questionnaire developed for this study. Responses were collected anonymously, and all 80 students completed the questionnaire. The three items measured overall satisfaction, perceived timeliness of feedback, and perceived specificity of feedback on a 5-point Likert scale. The questionnaire items were reviewed by clinical education experts for clarity and relevance before use. Internal consistency reliability in the present sample was acceptable, with a Cronbach’s α of 0.81. Because this was a brief study-specific instrument rather than a previously established validated scale, the satisfaction findings were interpreted cautiously.

Process indicators included the number of revisions, average practice frequency, and session duration, reflecting iterative learning behavior.

### 2.6 Sample size estimation

This study used a superiority design with the clinical reasoning score as the primary endpoint. A pilot study showed a standard deviation of about 1.5 points. Assuming a medium effect size (Cohen’s *d* = 0.5), α = 0.05 (two-sided), and 80% power, the required sample size was estimated at 34–36 per group. The final enrollment of 40 per group was therefore adequate.

### 2.7 Statistical analysis

All analyses were conducted using SPSS 25.0 and Python 3.9 (SciPy). Continuous variables were expressed as mean ± standard deviation, and categorical variables as frequencies and percentages.

Independent-sample *t*-tests were used for between-group comparisons when normality was satisfied, and paired-sample *t*-tests were applied for pre–post comparisons. For non-normal or ordinal variables such as Likert items, Mann–Whitney U tests were used where appropriate.

Normality was examined using the Shapiro–Wilk test, Q–Q plots, and skewness/kurtosis indices. With 40 participants per group, the central limit theorem supported approximate normality for major continuous variables. Homogeneity of variance was assessed before between-group comparisons. Effect sizes were reported as Cohen’s d and interpreted together with statistical significance. In addition to *P-*values, 95% confidence intervals (CIs) for the between-group differences were calculated and reported for the main outcomes. Inter-rater reliability for instructor-rated outcomes was assessed using the ICC. No formal adjustment for multiple comparisons was applied to subgroup or dimension-level analyses; therefore, these findings should be interpreted as exploratory and hypothesis-generating.

### 2.8 Ethics statement

This study was approved by the Ethics Committee of Peking University Third Hospital (No. IRB00006761-M2022063). All participants provided written informed consent. Data were anonymized and complied with ethical requirements for medical education research.

## 3 Results

### 3.1 Internal technical validation of the system

This section was included to demonstrate the technical feasibility of the system for supporting the educational intervention and was not treated as a primary study outcome. It served as an internal reliability check rather than part of the main between-group comparison.

The results showed that the multi-task model developed in this study performed well in dialogue generation, structured scoring accuracy, and feedback quality. The dialogue system generated responses within approximately 0.8 s per turn, which met the real-time requirements of the teaching system. The quality of the generated responses was acceptable to clinical instructors. The accuracy of the structured scoring across five dimensions exceeded 90%, and the F1-score was consistently above 0.87, indicating high scoring accuracy.

To further verify the relative advantage of system performance, mainstream models such as GPT-3.5, ChatGLM, Baichuan, and LLaMA2 were compared. Among the tested models, ChatGLM showed relatively balanced performance in scoring accuracy, structural recognition, and response efficiency for this teaching platform. Baichuan showed slightly better naturalness of text generation but weaker structural recognition.

### 3.2 Educational effects of combined training

At baseline, there were no significant differences between the experimental and control groups (*P* > 0.05). After the intervention, the experimental group showed greater improvement in clinical reasoning scores (1.3 ± 0.7 vs. 0.3 ± 0.5, 95% CI: 0.73–1.27, *P* < 0.01), core competencies scores (9.9 ± 4.1 vs. 3.8 ± 2.7, 95% CI: 4.56–7.64, *P* < 0.01), and OSCE performance (11.9 ± 5.2 vs. 3.2 ± 5.0, 95% CI: 6.43–10.97, *P* < 0.01) (Table 2). Inter-rater reliability was good for both the total clinical reasoning score (ICC = 0.87) and the total OSCE score (ICC = 0.84).

**TABLE 2:** Comparison of pre- and post-test results and teaching intervention effects among students.

Indicator	Experimental group Pre	Experimental group Post	Δ Experimental group	Control group Pre	Control group Post	Δ Control group	Difference in change (95% CI)	*P-*value*
Clinical reasoning score	5.9 ± 1.3	7.2 ± 1.0	1.3 ± 0.7	5.8 ± 1.2	6.1 ± 1.1	0.3 ± 0.5	0.73–1.27	< 0.01
Core competencies total score	72.1 ± 6.8	82.0 ± 5.6	9.9 ± 4.1	72.3 ± 7.1	76.1 ± 6.2	3.8 ± 2.7	4.56–7.64	< 0.01
- Professionalism	3.8 ± 0.6	4.3 ± 0.5	0.5 ± 0.4	3.9 ± 0.6	4.1 ± 0.5	0.2 ± 0.1	0.17–0.43	0.02
- Communication and collaboration	3.6 ± 0.7	4.4 ± 0.5	0.8 ± 0.4	3.7 ± 0.6	4.0 ± 0.6	0.3 ± 0.1	0.37– 0.63	< 0.01
- Teaching ability	3.4 ± 0.5	3.8 ± 0.5	0.4 ± 0.3	3.5 ± 0.6	3.7 ± 0.6	0.2 ± 0.1	0.10– 0.30	0.08
- Lifelong learning	3.5 ± 0.6	3.9 ± 0.5	0.4 ± 0.3	3.6 ± 0.6	3.8 ± 0.6	0.2 ± 0.1	0.10– 0.30	0.07
- Internal medicine knowledge and skills	3.7 ± 0.7	4.5 ± 0.5	0.8 ± 0.4	3.8 ± 0.6	4.1 ± 0.5	0.3 ± 0.1	0.37– 0.63	< 0.01
- Patient care	3.6 ± 0.6	4.4 ± 0.5	0.8 ± 0.4	3.7 ± 0.6	4.0 ± 0.5	0.3 ± 0.1	0.37– 0.63	< 0.01
OSCE score	71.2 ± 7.3	83.1 ± 6.1	11.9 ± 5.2	71.0 ± 7.4	74.2 ± 6.9	3.2 ± 5.0	6.43– 10.97	< 0.01

*P-value refers to the comparison of changes (Δ) between the experimental and control group.

Within the core competencies dimensions, the most significant improvements were observed in communication and collaboration (0.8 ± 0.4 vs. 0.3 ± 0.1, 95% CI: 0.37–0.63, *P* < 0.0*1*) and patient care (0.8 ± 0.4 vs. 0.3 ± 0.1, 95% CI: 0.37–0.63, *P* < 0.01). Professionalism and internal medicine knowledge and skills also improved moderately (*P* < 0.05). In contrast, teaching ability and lifelong learning showed slight increases that did not reach statistical significance (*P* > 0.05). The largest between-group differences were observed in communication and collaboration, patient care, and internal medicine knowledge and skills.

Students in the experimental group had a higher average number of weekly practice sessions (4.9 ± 1.3 per week) and longer individual practice durations (28.4 ± 6.5 min) compared with the control group, which reflected offline SPs training only (Table 3). The number of revisions was also significantly higher in the experimental group (3.8 ± 1.2 vs. 0.5 ± 0.3, 95% CI: 2.91–3.69, *P* = 0.03). Each revision led to an average improvement of 0.45 ± 0.18 points in clinical reasoning scores for the experimental group, compared with 0.12 ± 0.02 points in the control group (95% CI: 0.27–0.39, *P* < 0.01). The experimental group showed greater score improvement per revision than the control group.

**TABLE 3:** Students’ learning behaviors, satisfaction, and teachers’ grading efficiency.

Indicator	Experimental group	Control group	Difference in change (95% CI)	*T*-value	*P-*value	Effect size (Cohen’s *d*)
Average weekly practice frequency (times/week)	4.9 ± 1.3	1.1 ± 0.6	3.35–4.25	13.27	< 0.01	2.15
Average practice duration (minutes)	28.4 ± 6.5	17.9 ± 5.1	7.90–13.10	7.21	0.02	1.28
Number of revisions (times)	3.8 ± 1.2	0.5 ± 0.3	2.91–3.69	14.7	0.03	3.3
Average score improvement per revision	0.45 ± 0.18	0.12 ± 0.02	0.27–0.39	8.53	< 0.01	1.52
Improvement in students (< 6 points)	1.8 ± 0.6	0.4 ± 0.1	1.21–1.59	3.6	< 0.01	0.92
Improvement in students (≥ 6 points)	0.7 ± 0.3	0.3 ± 0.1	0.30–0.50	1.4	0.02	0.88
Overall student satisfaction	4.6 ± 0.5	3.2 ± 0.8	1.10–1.70	8.44	< 0.01	1.50
Feedback timeliness	4.5 ± 0.6	3.1 ± 0.9	1.06–1.74	8.19	< 0.01	1.83
Feedback specificity	4.4 ± 0.6	3.0 ± 0.7	1.11–1.69	9.60	< 0.01	2.15
Teacher grading time (minutes per case)	7.0 ± 1.2	15.0 ± 2.1	–8.76 to –7.24	14.83	< 0.01	-2.30

For the control group, practice frequency and duration refer only to the weekly SP interviews, while for the experimental group they include both VSP and SP sessions. Score improvement refers to the increase of clinical reasoning score from pre-test to post-test.

Subgroup analysis revealed that students with lower baseline scores (< 6 points) showed greater improvement (1.8 ± 0.6 vs. 0.4 ± 0.1, 95% CI: 1.21–1.59, *P* < 0.001, Cohen’s *d* = 0.92), whereas differences were smaller among students with higher baseline scores (≥ 6 points; 0.7 ± 0.3 vs. 0.3 ± 0.1, 95% CI: 0.30–0.50, *P* = 0.02, Cohen’s *d* = 0.88). Because no formal adjustment for multiple comparisons was applied, these subgroup findings should be interpreted as exploratory and hypothesis-generating. Overall, the effect size for clinical reasoning scores between groups was medium to large (Cohen’s d = 0.90).

Regarding transfer effects, OSCE scores in the experimental group improved from 71.2 ± 7.3 to 83.1 ± 6.1, while the control group improved from 71.0 ± 7.4 to 74.2 ± 6.9. The greatest improvement in the experimental group was seen in the history-taking station (*P* < 0.01).

### 3.3 Student and teacher feedback

Survey results showed that 92% of students believed the model’s feedback helped clarify diagnostic reasoning, 87% felt the system’s suggestions were timelier and more specific than offline feedback, and 81% reported that the system was suitable for repeated practice after class and could replace some traditional grading tasks. The 3-item learner-reported satisfaction questionnaire showed acceptable internal consistency in the present sample (Cronbach’s α = 0.81). Learner-reported satisfaction was significantly higher in the experimental group (4.6 ± 0.5 vs. 3.2 ± 0.8, 95% CI: 1.10–1.70, *P* < 0.01). The experimental group also outperformed the control group in both feedback timeliness and feedback specificity.

However, several students suggested improvements. Some reported that the AI feedback tended to be “templated” and lacked clinical nuance. In cases where AI scoring and teacher scoring were inconsistent, students experienced confusion and sometimes reduced confidence. In addition, some instructors expressed concerns that excessive reliance on AI and immediate feedback might reduce students’ tolerance for uncertainty and weaken their independent reasoning ability.

Teacher feedback indicated that the system reduced the time required for manual grading from about 15.0 ± 2.1 min per case to 7.0 ± 1.2 min, cutting workload by approximately 53%. Teacher grading time per case was shorter in the experimental group than in the control group. Nonetheless, teachers emphasized that AI still lagged behind human experts in understanding complex patient histories, and that instructor oversight remained essential in critical steps.

## 4 Discussion

The findings of this study indicate that the combined training model using VSPs (AI systems) and SPs was associated with better educational outcomes than the SPs-only training approach used in this cohort. The results suggested that the combined approach was associated with greater improvement in clinical reasoning scores and history-taking performance, with transfer to objective examinations such as the OSCE.

The advantages of this model lie in its complementarity. While SPs provide authentic clinical interaction and communication scenarios, the introduction of AI systems into the combined training model offers high-frequency practice and immediate feedback, addressing the limitations of SPs in terms of personalization and repeatability.

From an educational perspective, the study found that students with lower baseline scores benefited more, suggesting that this model helps narrow performance gaps among students and thus promotes educational equity. At the same time, the automatic scoring function of the AI system reduced faculty workload, allowing instructors to focus more on higher-order reasoning and individualized guidance, thereby optimizing the use of teaching resources.

### 4.1 Technical performance and system advantages

The deep learning–based training system developed in this study demonstrated educational value and technical advantages. The model achieved high accuracy in scoring, recognition of structural dimensions, and generation of textual feedback. The consistency between automatic and expert scoring highlighted the potential of reliable automated evaluation. Intervention results further showed that students in the combined training group improved more in clinical reasoning than those in the SPs-only group, suggesting that the combined training approach may have supported improvements in reasoning structure and logical expression.

Previous studies also support the potential of large language models in medical education. For example, Schaye et al. developed an LLM model to score clinical reasoning records in electronic health records, achieving good agreement with expert ratings and confirming the feasibility of AI-assisted reasoning assessment (11). Cianciolo et al. similarly demonstrated that machine scoring could serve as a useful complement to instructor evaluation (12).

In our internal model comparison, ChatGLM showed a relatively balanced performance in scoring accuracy, reasoning structure recognition, feedback language quality, and response speed for real-time educational use (13). Baichuan achieved slightly higher BLEU scores but had weaker structural recognition and longer response times, while GPT-3.5 was stable but consumed more computational resources, increasing deployment costs.

### 4.2 Educational value and learning outcomes

Further analysis suggested that the structured feedback and revision process within the combined training approach may have contributed to improved student outcomes. This closed-loop chain of diagnosis, scoring, feedback, and revision enhanced training frequency, engagement, and the ability to identify reasoning gaps and repair logic chains.

Beyond clinical reasoning scores, the experimental group also showed significant improvement in overall core competencies. Gains were particularly notable in communication, patient care, and knowledge and skills, suggesting that the system contributed to the development of broader professional competencies. These findings extend the educational application of AI systems from cognitive reasoning to practical competence, supporting the transfer from “thinking” to “behavior” in medical education (14).

Taken together, the results of model comparisons and feedback analyses confirm that this system possesses both technical robustness and educational utility. It may be recommended as part of standard teaching evaluation tools, particularly in institutions with limited resources or a need for large-scale training. However, limitations remain. When dealing with complex patient histories, ambiguous expressions, or emotional nuances, AI feedback sometimes appeared overly positive and lacked in-depth correction of key logical errors (15).

### 4.3 Applicability of structured clinical reasoning modeling

The system incorporated five core dimensions into a structured labeling framework. Results showed that the model effectively identified missing elements in students’ reasoning processes. This structured approach helped students detect and correct “blind spots” in reasoning, enhancing the completeness and coherence of their diagnostic logic. Previous studies have also emphasized the importance of structural labeling, particularly the logical connections between premises, evidence, conclusions, and management plans, in ensuring the quality of reasoning (16). By shifting from simple scoring to structured feedback, this system provided more instructive educational guidance, though its capacity to process implicit reasoning and cross-disciplinary knowledge integration remains limited and requires further improvement.

### 4.4 Individual differences and educational significance

Subgroup analysis revealed that students with lower baseline scores (< 6 points) improved substantially more, indicating that the system is particularly beneficial for weaker students and contributes to educational equity. Students with higher baseline scores also improved, though to a lesser extent. This suggests that future system development should incorporate stratified feedback modules to provide personalized learning pathways.

However, concerns remain regarding students’ potential overreliance on AI, which may weaken their independent reasoning and tolerance for uncertainty (17). Notably, the “teaching ability” and “lifelong learning” dimensions showed upward trends but did not reach statistical significance. This may be due to the short intervention period and the system’s focus on diagnostic logic and communication skills. Future enhancements could include teaching demonstration modules, learning trajectory tracking, and interdisciplinary task scenarios to foster lifelong learning awareness.

### 4.5 Transfer and retention of educational effects

The benefits of the intervention were not limited to simulated consultations but also transferred to objective examinations. OSCE scores in the experimental group increased, particularly in history-taking and justification, indicating that the combined intervention may have supported students’ ability to apply skills in objective examination settings. Prior studies support such transfer effects, showing that AI-powered VSPs can produce learning outcomes comparable to, or even better than, traditional SPs, highlighting their potential in large-scale medical education (18).

### 4.6 Faculty workload and future prospects

Faculty feedback revealed that the system reduced grading time, cutting workload by more than half. This human–AI collaborative model allowed instructors to focus on guiding higher-order reasoning while delegating routine grading tasks to AI, thereby improving efficiency and resource utilization. Previous research has shown that blended AI–human assessment systems can achieve both efficiency and reliability, offering new solutions for medical education (19–21).

The deep learning–based training system developed in this study may improve short-term educational outcomes and serve as a useful adjunct to SPs-based training and holds broad promise for teaching applications. It is recommended that medical education authorities consider incorporating such systems into standard curricula, especially in contexts such as online training during pandemics. Nonetheless, instructors stressed that AI should be positioned as a supportive tool rather than a full replacement, to avoid undermining the essential role of faculty in fostering clinical reasoning (22).

### 4.7 Limitations and future directions

Several limitations should be noted. First, the intervention group had more practice opportunities and feedback than the control group, which may have contributed to the observed differences. Second, this was a single-center study with a moderate sample size, and full blinding was difficult in this educational setting. Third, satisfaction outcomes were based on a brief study-specific self-reported questionnaire rather than a previously established validated scale, and may therefore have been influenced by response bias or Hawthorne effect. Fourth, subgroup and dimension-level analyses were exploratory and were not adjusted for multiple comparisons, which may increase the risk of type I error. Finally, AI-generated feedback may contain over-standardized content or occasional inaccuracies, and long-term follow-up was not available.

Therefore, the present study should be interpreted as supporting the effectiveness of the combined training package used in this study, rather than isolating the specific effect of the VSPs component alone.

Future research should involve multi-center, multi-cohort studies to verify system adaptability, while exploring stratified feedback, personalized training, and long-term follow-up to enhance the educational value of AI-assisted systems.

## 5 Conclusion

This study suggests that combined training using AI-powered VSPs and SPs was associated with better short-term educational outcomes than the SPs-only training approach used in this study.

## Data Availability

The original contributions presented in the study are included in the article/supplementary material, further inquiries can be directed to the corresponding authors.
